# Treatment combining aliskiren with paricalcitol is effective against progressive renal tubulointerstitial fibrosis via dual blockade of intrarenal renin

**DOI:** 10.1371/journal.pone.0181757

**Published:** 2017-07-28

**Authors:** Sungjin Chung, Soojeong Kim, Minyoung Kim, Eun Sil Koh, Seok Joon Shin, Cheol Whee Park, Yoon Sik Chang, Ho-Shik Kim

**Affiliations:** 1 Department of Internal Medicine, College of Medicine, The Catholic University of Korea, Seoul, Republic of Korea; 2 Division of Nephrology and Hypertension, Department of Medicine, Vanderbilt University School of Medicine, Nashville, Tennessee, United States of America; 3 Department of Biochemistry, College of Medicine, The Catholic University of Korea, Seoul, Republic of Korea; University of Louisville, UNITED STATES

## Abstract

The aim of this study was to assess any potential additive effects of a treatment combining aliskiren with paricalcitol on reducing renal fibrosis. C57BL/6J mice were treated individually with aliskiren and/or paricalcitol until 7 days after initiation of unilateral ureteral obstruction (UUO).In obstructed kidneys of UUO mice, monotherapy with aliskiren or paricalcitol significantly attenuated interstitial fibrosis, collagen IV accumulation, and α-smooth muscle actin- and terminal deoxynucleotidyl transferase-mediated biotin nick end-labeling-positive cells. The combination treatment showed additive efficacy in inhibition of these parameters. Renal NADPH oxidase (Nox)1 and Nox2 were significantly decreased by aliskiren or paricalcitol alone or in combination, while renal Nox4 expression was significantly reduced by paricalcitol mono- or combination treatment. Increased levels of p-Erk and p-p38 MAPK, and NF-κB in UUO kidneys were also significantly reduced by either aliskiren or paricalcitol treatment alone or in combination. Aliskiren or paricalcitol monotherapy significantly reduced the expression of (pro)renin receptor in UUO kidneys. In addition, aliskiren tended to augment renin expression in UUO kidneys, but paricalcitol reduced its expression level. The combination treatment effectively blocked both (pro)renin receptor and renin expression induced by aliskiren, and resulted in a further reduction of the renal expression of angiotensin II AT1 receptor. Aliskiren failed to increase the expression of vitamin D receptor in UUO kidneys, but the combination treatment restored its expression level. Taken together, a treatment combining aliskiren with paricalcitol better inhibits UUO-induced renal injury. The mechanism of this synergy may involve more profound inhibition of the intrarenal renin-angiotensin system.

## Introduction

Renin is synthesized by the juxtaglomerular cells of the renal afferent arterioles as preprorenin [1]. Cleavage of a 23 amino acid peptide at the carboxyl terminus of preprorenin generates prorenin, which is then converted to active renin by proteolytic cleavage of the 43-amino acid N-terminal prosegment [1–2]. Renin acts in the rate-limiting step to cleave angiotensinogen synthesized by the liver, thus forming angiotensin I [3–4]. Angiotensin-converting enzyme (ACE) then converts angiotensin I to angiotensin II, and it also degrades bradykinins to inactive fragments, reducing the serum levels of endogenous vasodilators [4]. Thus, renin is positioned at the top of the renin-angiotensin system (RAS) that preserves end-organ perfusion by regulating extracellular fluid, sodium and water balance, and cardiovascular activity [4–5]. In various renal injury models, the activation of the RAS increases transforming growth factor-beta (TGF-β), plasminogen activator inhibitor (PAI)-1 and α-smooth muscle actin (α-SMA) expressions in the kidneys [1, 6], contributing to the progression of renal interstitial fibrosis.

Under physiologic conditions, renin produced by the juxtaglomerular cells is sufficient to maintain blood pressure, intravascular volume and glomerular filtration rate [3]. When pathological conditions occur, additional renin-producing cells, such as pericytes and vascular smooth muscle cells, are recruited extramurally within the interstitium [3, 7]. When interstitial fibrosis develops due to chronic progressive renal injury, the interstitium is occupied predominantly by extracellular matrix (ECM), ECM-producing fibroblasts, and inflammatory cells [3, 8], and renin-producing cells also accumulate in the fibrotic interstitium, while erythropoietin-producing fibroblasts are lost [3]. According to recent studies, renin-producing cells are rather plastic and may transform into other renal cell types, such as myofibroblasts, and thus they significantly contribute to renal fibrosis [9]. Therefore, additional recruitment of renin-producing cells and subsequent increase of intrarenal renin expression appear to be associated with chronic kidney disease (CKD).

On the other hand, the binding between (pro)renin and (pro)renin receptor (PRR) induces prorenin enzymatic activation without cleavage of the prosegment, and triggers intracellular signaling pathways independent of the RAS [1]. Experimental evidence has shown that (pro)renin and PRR participate in the development of renal fibrosis. In human kidney (HK)-2 cells, PRR knock-down inhibits indoxyl sulfate-induced expression of TGF-β1 and α-SMA, and production of reactive oxygen species and activation of Stat3 and nuclear factor kappa B (NF-κB) are associated with upregulation of (pro)renin and PRR [10]. Additionally, the activation of PRR by (pro)renin induces expression of PAI-1, fibronectin and collagen I[11], and it also stimulates mitogen-activated protein kinase (MAPK) signaling [12]. Furthermore, PRR expression was reported to be upregulated in the renal tubules of an animal model of CKD [10]. Thus, pro(renin) and PRR are involved in the development of renal fibrosis through multiple RAS-independent signaling pathways.

It is apparent that renin plays a central role in the process of renal tubulointerstitial fibrosis. Maximally reducing renin expression and its activity in diseased kidneys will help protect kidneys from progressing to advanced CKD. Aliskiren is an oral direct renin inhibitor that has been introduced to treat hypertension [6]. Subsequently, independent of blood pressure control, aliskiren alone or in combination with other drugs has been shown to attenuate renal fibrosis and inflammation induced by unilateral ureteral obstruction (UUO) [6, 13]. In a mouse model of UUO, aliskiren decreased the increased levels of inflammatory markers such as renal CD68-positive cells, monocyte chemotactic protein (MCP)-1 and osteopontin mRNA levels in obstructed kidneys [6]. Aliskiren treatment in a rat model of reversible UUO resulted in an attenuation of gene expression of markers of renal injury such as neutrophil gelatinase-associated lipocalin, kidney injury molecule-1 and p53 [14]. Compared with an ACE inhibitor, there was no difference in decreasing albuminuria and glomerulosclerosis in an animal model of diabetes, but the magnitude of interstitial fibrosis was attenuated to a greater degree by aliskiren than by an ACE inhibitor [15]. There have also been some reports that show the possible synergistic effect of combination therapy with aliskiren and other drugs. A study demonstrated that combination therapy with aliskiren and mizoribine could result in increased renal protection against fibrosis and inflammation as evidenced by attenuation of tubulointerstitial changes, and decrease of α-SMA expression, monocyte and macrophage infiltration and TGF-β1 mRNA expression in UUO-induced kidneys [13]. It seems to be apparent that aliskiren in conjunction with an angiotensin II receptor blocker can provide increased renal protection against renal injury during obstruction over either agent alone [16]. Another study has shown that in obstructed kidneys of Toll-like receptor 2-knockout mice, aliskiren further decreased the renal tissue injury score and mRNA expression of MCP-1, osteopontin and TGF-β [17].

Although aliskiren decreases the plasma renin activity and angiotensin II activity, it tends to induce a reactive increase in renin secretion and expression in the kidney, leading to activation of fibrotic signaling pathways mediated by PRR [6]. Vitamin D or its analogue may help remedy this imperfection. Vitamin D, known for its pleiotropic effects, reduces activation of the intrarenal RAS by acting as a negative regulator of renin gene expression [18], and inhibits renal inflammation and fibrosis, at least partly by targeting the RAS [19]. In an obstructive nephropathy model, paricalcitol, a vitamin D analogue, was able to ameliorate renal interstitial fibrosis [19]. A study group has proposed that the mitochondrial nongenomic activity of vitamin D receptor (VDR) could be associated with RAS system counterbalance and such activation of VDR by paricalcitol has an angiotensin II AT1 receptor (AT1R)-dependent protective effect at the mitochondrial level [20]. On the other hand, combination therapy with paricalcitol and an ACE inhibitor demonstrated additive efficacy in retarding renal scar formation during obstruction nephropathy [21].

The mechanisms responsible for renal fibrosis are multifactorial and complex [13, 16, 21]. Therefore, combination treatment with different classes of agents may be a rational strategy for CKD treatment [16, 21]. Since aliskiren and vitamin D suppress renin through different mechanisms, we hypothesized that the combination treatment with aliskiren and paricalcitol could be more effective in inhibiting renin expression in the diseased kidney. This study investigated the possible role of this combined treatment in UUO-induced renal tubulointerstitial fibrosis.

## Materials and methods

### Animal experiment

The study was approved by The Institutional Animal Care and Use Committee of The Catholic University of Korea Yeouido St. Mary’s Hospital (No. YEO20141702FA). Aliskiren used in this study was provided by Novartis Pharma AG (Basel, Switzerland) after screening the experimental protocol (NIBR-MTM 37399). Male C57BL/6J mice weighing 20–25 g (DooYeol Biotech., Seoul, Republic of Korea) were randomized into the following five groups: (1) sham group (Sham; n = 6); (2) UUO control group (UUO; n = 8) treated with olive oil (Sigma-Aldrich, St. Louis, MO, USA) as a vehicle; (3) UUO group (UUO+A; n = 8) treated with aliskiren 20 mg/kg/day via a mini-osmotic pump (Alzet Osmotic Pumps, Cupertino, CA, USA); (4) UUO group (UUO+P; n = 8) treated by daily intraperitoneal injection of paricalcitol (Abbott, IL, USA) at a dose of 0.3 μg/kg/day; and (5) UUO group (UUO+A+P; n = 8) treated with both aliskiren and paricalcitol. All treatments were administered one day before UUO, and each treatment was continued for 7 days after surgery. The dose of aliskiren chosen in this study was expected to be non-hypotensive, based on prior data [6, 16]. Blood pressure of mice was measured by tail-cuff plethysmography (IITC Life Science, Woodland Hills, CA, USA) as described previously [22], and the measurement confirmed that systolic blood pressure was similar among groups on day 6 after UUO (data not shown). UUO was performed as described previously [22–23]: general anesthesia was induced by inhalation of isoflurane mixed with air using a vaporizer. After routine sterilization and shaving, the abdomen was opened through a left incision and the left ureter was located and occluded at two places at the level of the lower pole of the left kidney with non-absorbable 4–0 silk. The abdomen was closed with a running suture and the skin was closed with interrupted sutures. Sham operation was performed by using identical surgical procedures without ureteral ligation. The total operation time was approximately 10 min. After UUO operation, 1 mL of 0.9% saline, warmed prior to injection, was administered intraperitoneally for maintaining hydration status and then mice were kept warm until they had completely recovered. The steps for assessing clinical signs of pain and distress included gross inspection of animals for abnormalities in appearance and behavior, lifting the cage wire lid to elicit a response, and examination of each individual mouse by gently restraining the animal. For daily monitoring, the following parameters were used: attitude, gait and posture, body weight and food intake. During the whole study period, mice were maintained under specific pathogen-free conditions in isolated cages with a 12 h light / 12 h dark photoperiod in a humidity- and temperature-controlled room. Water and food were available ad libitum. No mortality was observed in any group. On day 7 after the UUO operation, mice were sacrificed by exsanguination and cervical dislocation under isoflurane anesthesia. After perfusion with cold phosphate-buffered saline (PBS), the obstructed left kidneys were rapidly removed, cut along the coronal plane, fixed in a periodate-lysine-paraformaldehyde solution, processed and embedded in paraffin. The remaining renal cortex was snap-frozen in liquid nitrogen for further molecular measurements.

### Histological and immunohistochemical analyses

After deparaffinization of the obstructed left kidney samples, 4 μm sections were processed and stained with Masson’s trichrome and α-SMA stain as reported previously [22–23]. Tubulointerstitial fibrosis, assessed on sections stained by trichrome (ScyTek Laboratories, Logan, UT, USA), was defined as a matrix-rich expansion of the interstitium with tubular dilatation, atrophy, cast formation, and sloughing of tubular epithelial cells or thickening of the tubular basement membrane. The matrix score for expression of myofibroblasts in the renal cortical interstitium was determined using the monoclonal antibody against α-SMA (Abcam, Cambridge, MA, USA). At least 10 fields per section were assessed in a blinded manner by counting the percentage of injured areas per field of cortex at x 200 magnification.

### Terminal deoxynucleotidyl transferase-mediated biotin nick end-labeling (TUNEL) assay

Apoptosis in renal tissues was assessed using TUNEL assay as described previously [22, 24]. Detection of apoptotic cells in paraffin-embedded tissue was performed using the S7101 ApopTag Plus peroxidase in situ apoptosis detection kit according to the manufacturer’s instructions (Merck Millipore, Darmstadt, Germany). TUNEL-positive cells were evaluated in 10 randomly selected tubulointerstitial fields of each kidney sample at x 200 magnification.

### Western blotting

The total cellular protein content of renal tissues was extracted as described previously [22–24]. Briefly, equal amounts of proteins were electrophoresed by sodium dodecyl sulfate-polyacrylamide gel electrophoresis and transferred to nitrocellulose membranes (Bio-Rad, Hercules, CA, USA). Nonspecific binding was blocked with 5% skim milk. Filters were incubated overnight at 4°C with antibodies directed against the following proteins: type IV collagen (Col IV; Abcam); Nox1 (Santa Cruz Biotechnology, Santa Cruz, CA, USA); Nox2 (BD Biosciences, San Jose, CA, USA); Nox4 (Santa Cruz Biotechnology); MAPKs, including extracellular signal-regulated kinase (Erk; Cell Signaling Technology, Beverly, MA, USA), phosphorylated Erk1/2 (p-Erk1/2; Cell Signaling Technology), p38 MAPK (p38; Cell Signaling Technology), and phosphorylated p38 MAPK (p-p38; Cell Signaling Technology); total NF-κB (Santa Cruz Biotechnology); phosphorylated NF-κB (p-NF-κB; Santa Cruz Biotechnology); PRR (Sigma-Aldrich); renin (Santa Cruz Biotechnology); AT1R (Santa Cruz Biotechnology); VDR (Santa Cruz Biotechnology); and β-actin (Sigma-Aldrich). After a wash, the blots were incubated with a secondary antibody conjugated with horseradish peroxidase. The protein bands were detected by enhanced chemiluminescence reagents and imaged using an Image Quant LAS 4000 (GE Healthcare, Piscataway, NJ, USA). The density of the bands was analyzed using ImageJ 1.49 software (National Institutes of Health, Bethesda, MD, USA).

### Statistical analysis

All values are expressed as the mean ± SE. Multiple comparisons among groups were performed by one-way analysis of variance with Bonferroni correction using SPSS 22.0.0.1 (IBM Corp., Armonk, NY, USA). Statistical significance was set at P<0.05.

## Results

### Renal tubulointerstitial fibrosis

**Fig 1:**
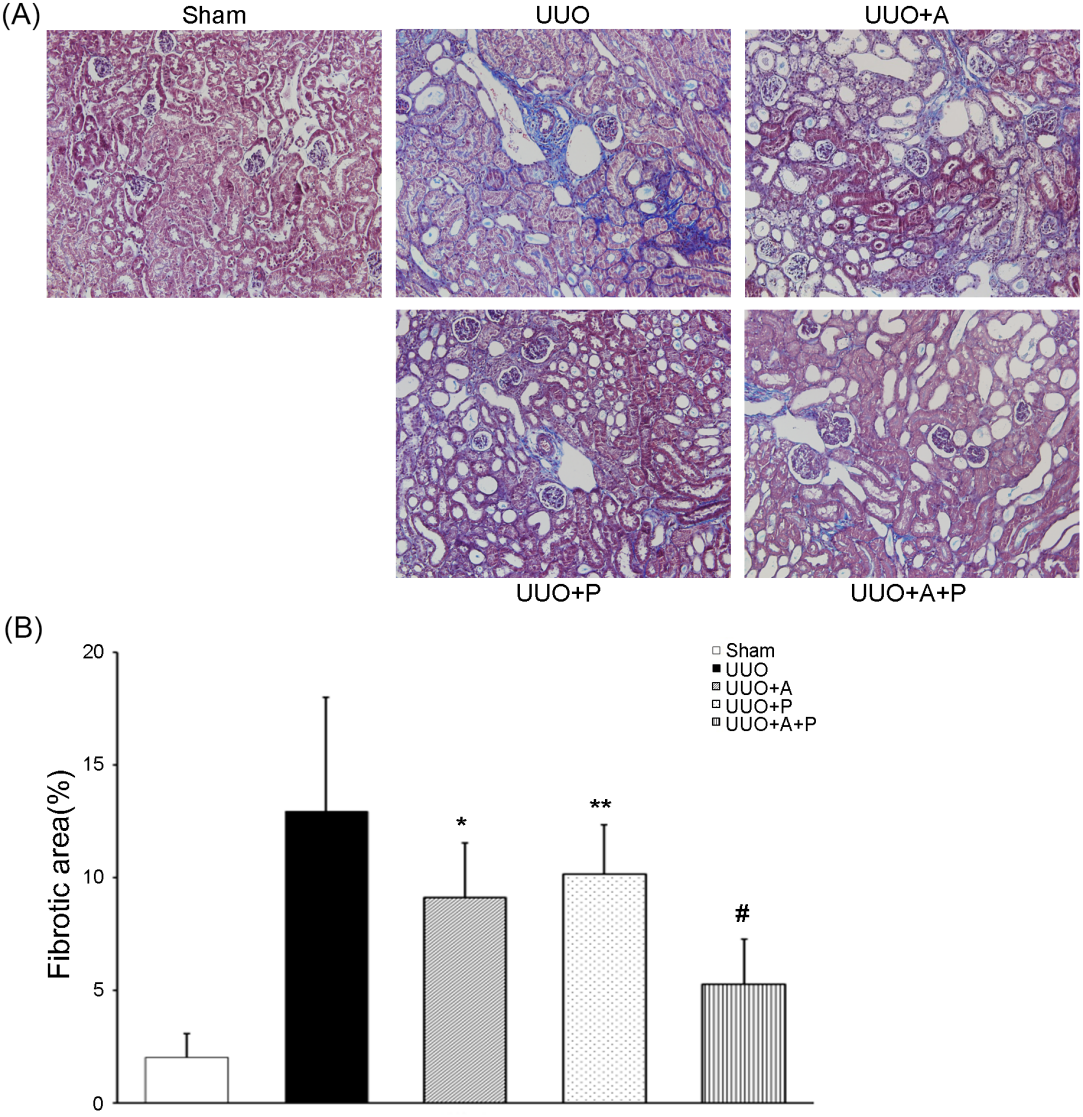
Greater beneficial effect of combination treatment with aliskiren and paricalcitol on renal fibrosis in the obstructed kidney. (A) Representative renal sections stained with Masson-trichrome (original magnifications, x200). (B) Quantitative analysis of the results for fibrotic area in the renal tubulointerstitium. *P<0.001 versus UUO; **P = 0.001 versus UUO; ^#^P<0.001 versus UUO+A or UUO+P.

Masson’s trichrome staining revealed the presence of tubulointerstitial fibrosis in the UUO groups compared with the Sham group (Fig 1A). However, the degree of tubulointerstitial fibrosis in the UUO+A group or the UUO+P group was significantly lower than that in the UUO control group. In the UUO+A+P group, there was a further significant decrease in tubulointerstitial fibrosis compared with the UUO+A group or the UUO+P group (Fig 1B).

**Fig 2:**
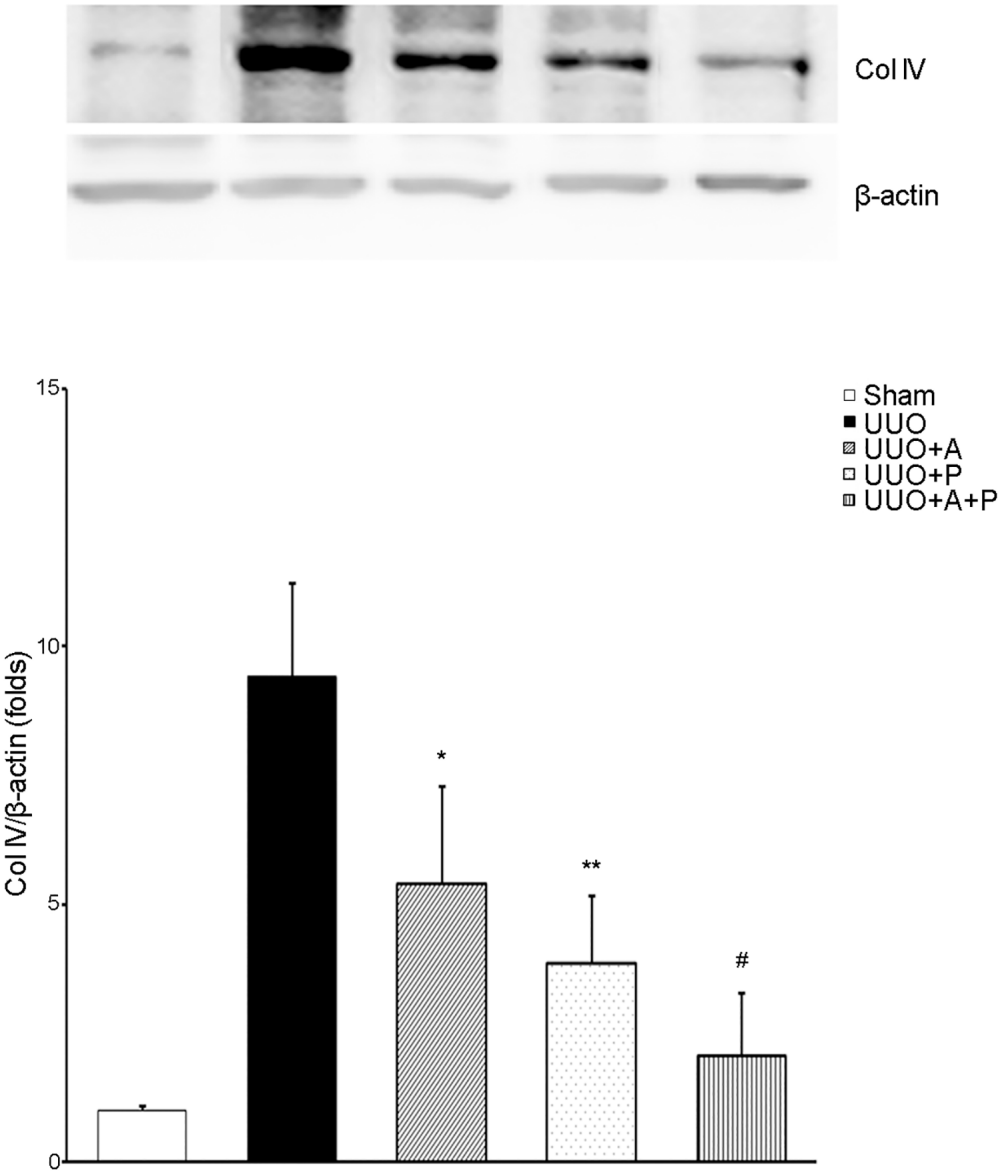
Efficacy of combination treatment with aliskiren and paricalcitol on tubulointerstitial expression of collagen IV in the obstructed kidney. Representative Western blot (upper) and its quantitative analysis (lower) showing Col IV expression in obstructive nephropathy. *P = 0.010 versus UUO; **P = 0.022 versus UUO; ^#^P = 0.031 versus UUO+A and P = 0.043 versus UUO+P. Col IV, type IV collagen.

The obstruction induced by UUO significantly increased the expression of Col IV (Fig 2).

However, aliskiren monotherapy significantly alleviated Col IV accumulation in UUO kidneys, and paricalcitol monotherapy more significantly decreased the Col IV expression. Expression of Col IV was further reduced when mice were treated with a combination of aliskiren and paricalcitol.

### Renal tubulointerstitial myofibroblast expression

**Fig 3:**
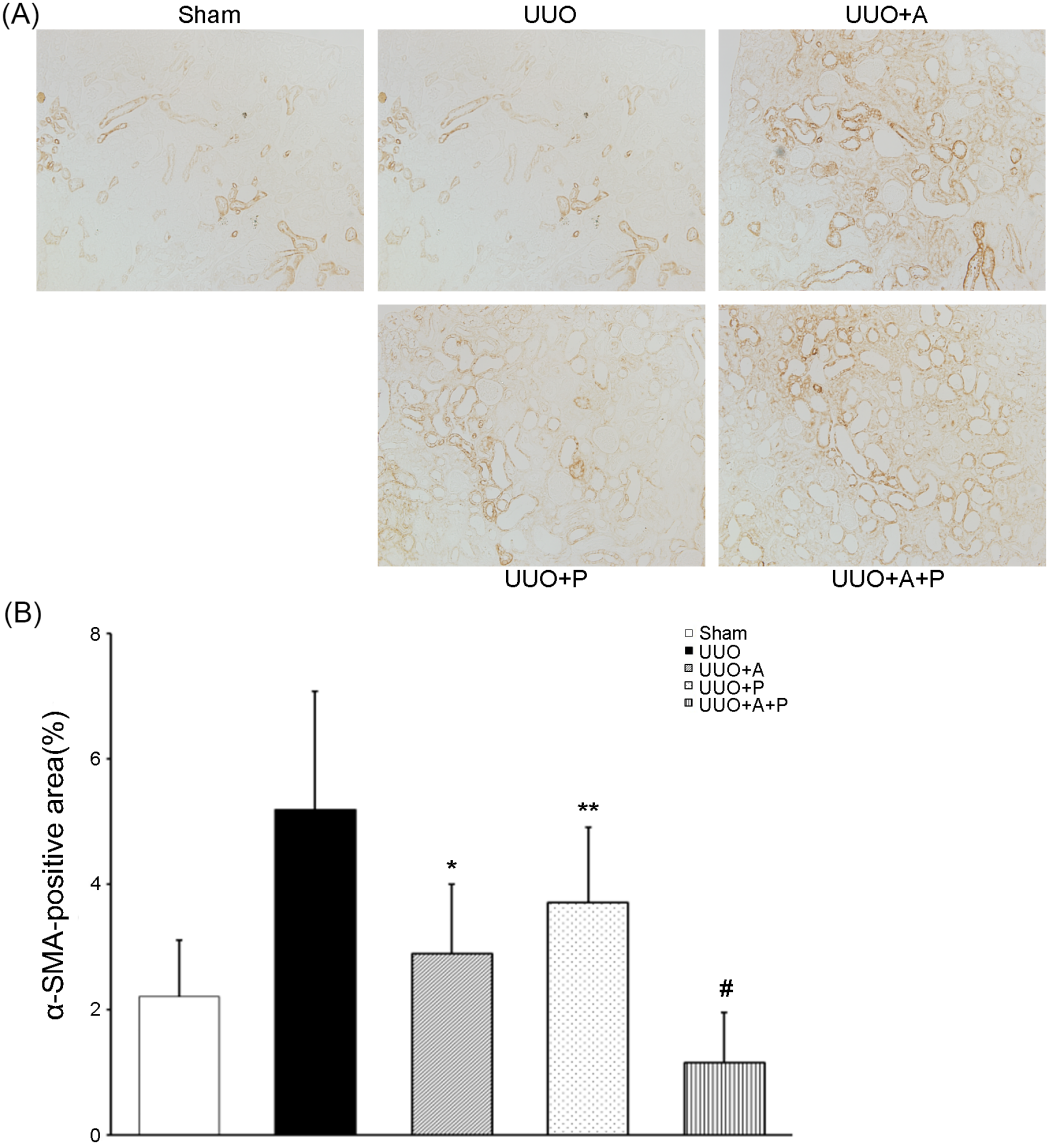
Synergistic effect of combination treatment with aliskiren and paricalcitol on the expression of myofibroblasts in the obstructed kidney. (A) Representative renal sections stained with α-SMA(original magnification, x200). (B) Quantitative analysis of the results for α-SMA in the renal tubulointerstitium. *P<0.001 versus UUO; **P = 0.001 versus UUO; ^#^P = 0.001 versus UUO+A and P<0.001 versus UUO+P. α-SMA, α-smooth muscle actin.

After UUO, α-SMA-positive kidney myofibroblasts were significantly increased in the obstructed kidneys (Fig 3A). In contrast, treatment with aliskiren or paricalcitol resulted in a decrease of α-SMA expression. Especially, combined use of aliskiren and paricalcitol in the UUO+A+P group caused most marked decrease in α-SMA-positive cells (Fig 3B).

### Renal tubulointerstitial apoptosis

**Fig 4:**
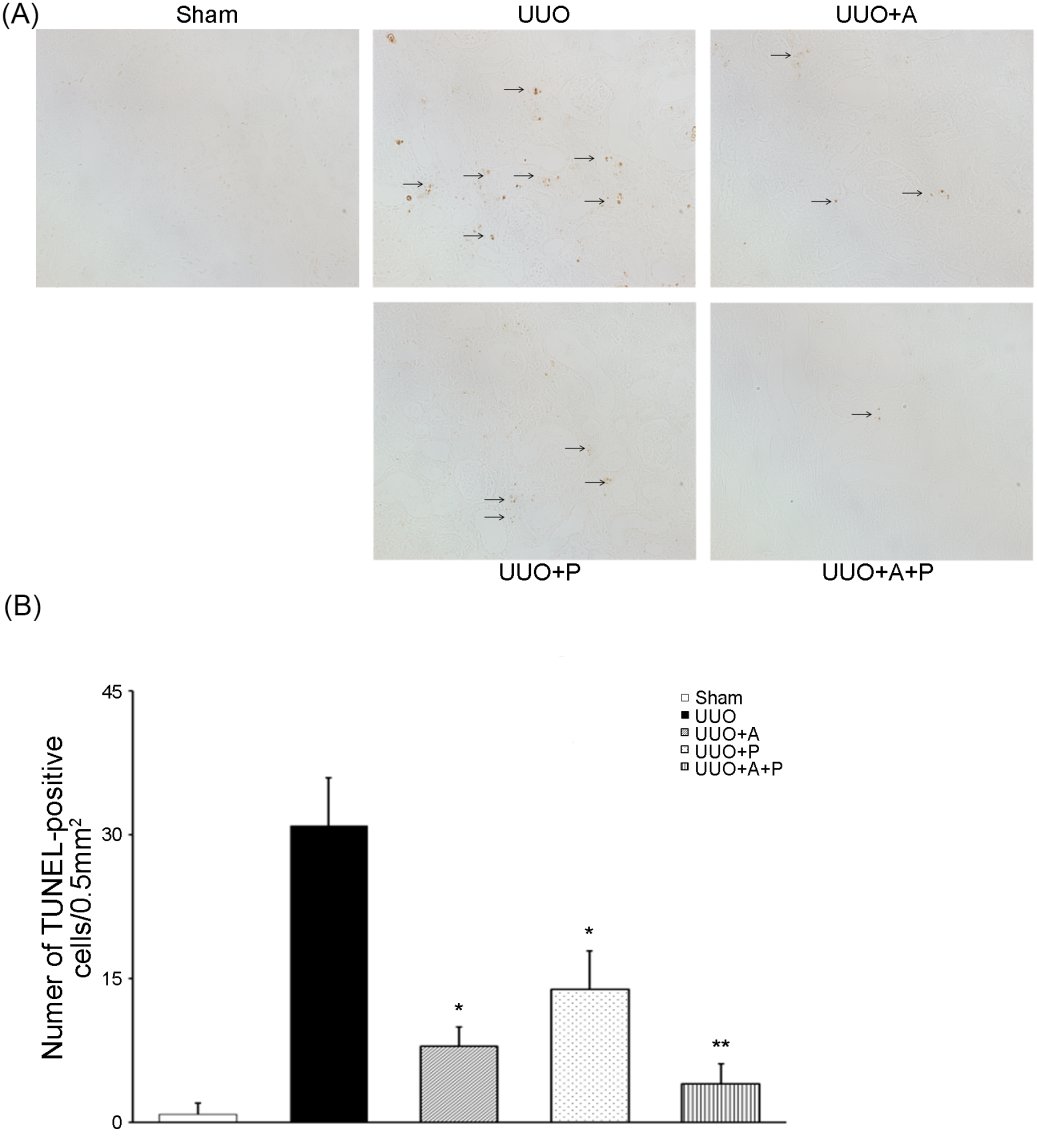
Additive effect of combination treatment with aliskiren and paricalcitol on apoptosis in the obstructed kidney. (A) Representative renal sections stained with TUNEL (original magnification, x200). (B) Quantitative analysis of the results for TUNEL assay in the renal tubulointerstitium. *P<0.001 versus UUO; **P = 0.021 versus UUO+A. TUNEL, terminal deoxynucleotidyl transferase-mediated biotin nick end-labeling.

To assess apoptosis in the kidney, TUNEL staining was performed. The number of TUNEL-positive cells was significantly higher in the UUO groups compared with the Sham group (Fig 4A). Administration of aliskiren or paricalcitolin the UUO-operated mice significantly reduced the number of TUNEL-positive cells by more than 50% compared with that in the UUO control group, whereas the treatment combining both aliskiren and paricalcitol in the UUO+A+P group resulted in the fewest number of TUNEL-positive cells when compared to that in the UUO+A group or the UUO+P group (Fig 4B).

### Renal oxidative stress

**Fig 5:**
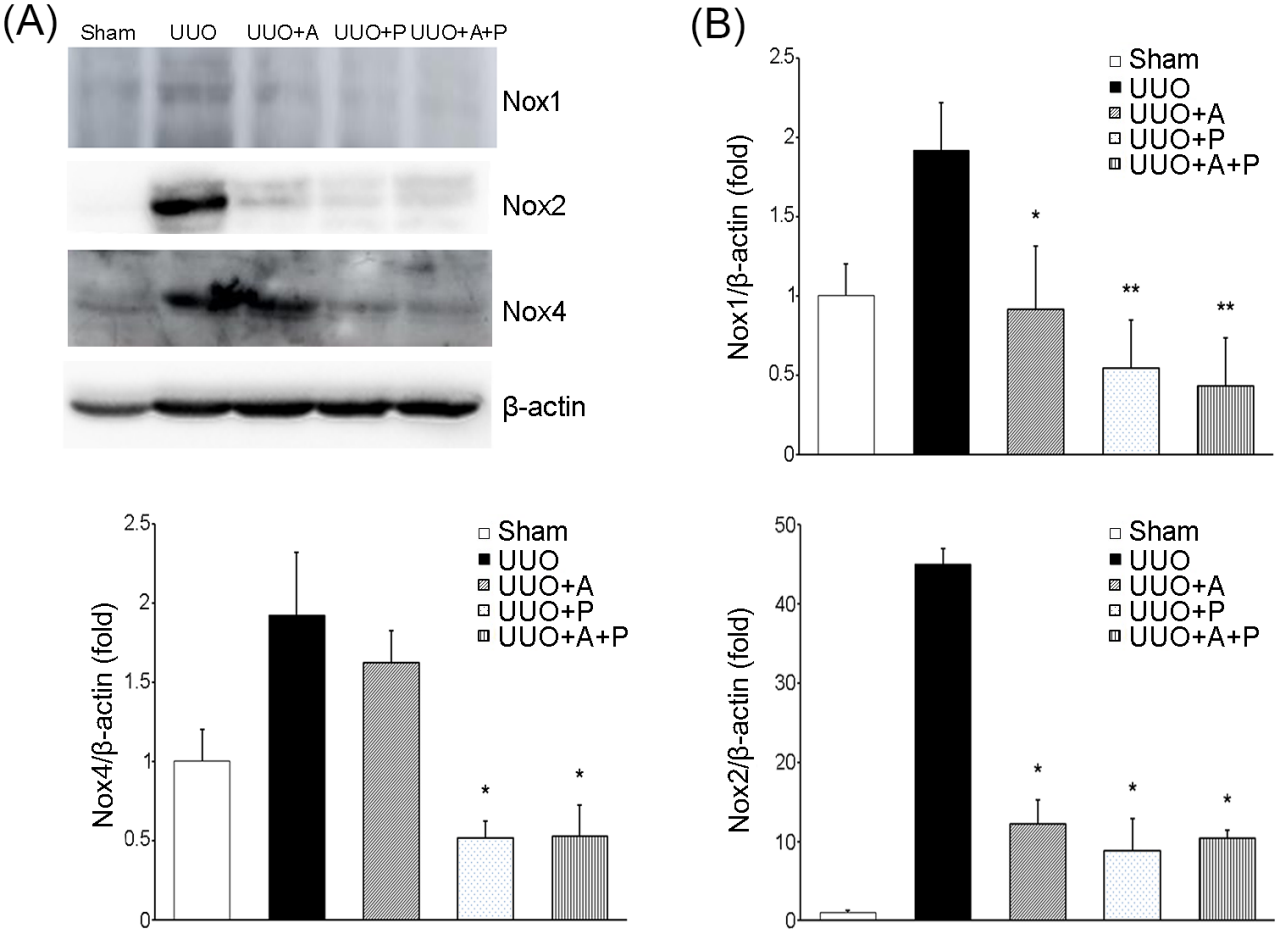
Effects of aliskiren or paricalcitol monotherapy or combination treatment with both drugs on NADPH oxidase isoforms in the obstructed kidney. (A) Representative immunoblot showing the expression of Nox1, Nox2, and Nox4. (B) Quantitative analysis for the expression of Nox1 (upper), Nox2 (lower right), and Nox4 (lower left). *P<0.001 versus UUO; **P = 0.001 versus UUO+A (for Nox1). *P <0.001 versus UUO (for Nox2).*P<0.001 versus UUO and UUO+A (for Nox4).

The obstruction induced by UUO caused an increased expression of Nox1, Nox2, and Nox4 (Fig 5). Aliskiren significantly prevented UUO-induced increase of Nox1 and Nox2, but it did not affect the expression of Nox4. On the other hand, the expressions of Nox1, Nox2 and Nox4 were significantly decreased by paricalcitol. Combination treatment of aliskiren and paricalcitol reduced the expressions of Nox1, Nox2, and Nox4 to levels similar to those following paricalcitol monotherapy.

### Mediators of the inflammatory response

**Fig 6:**
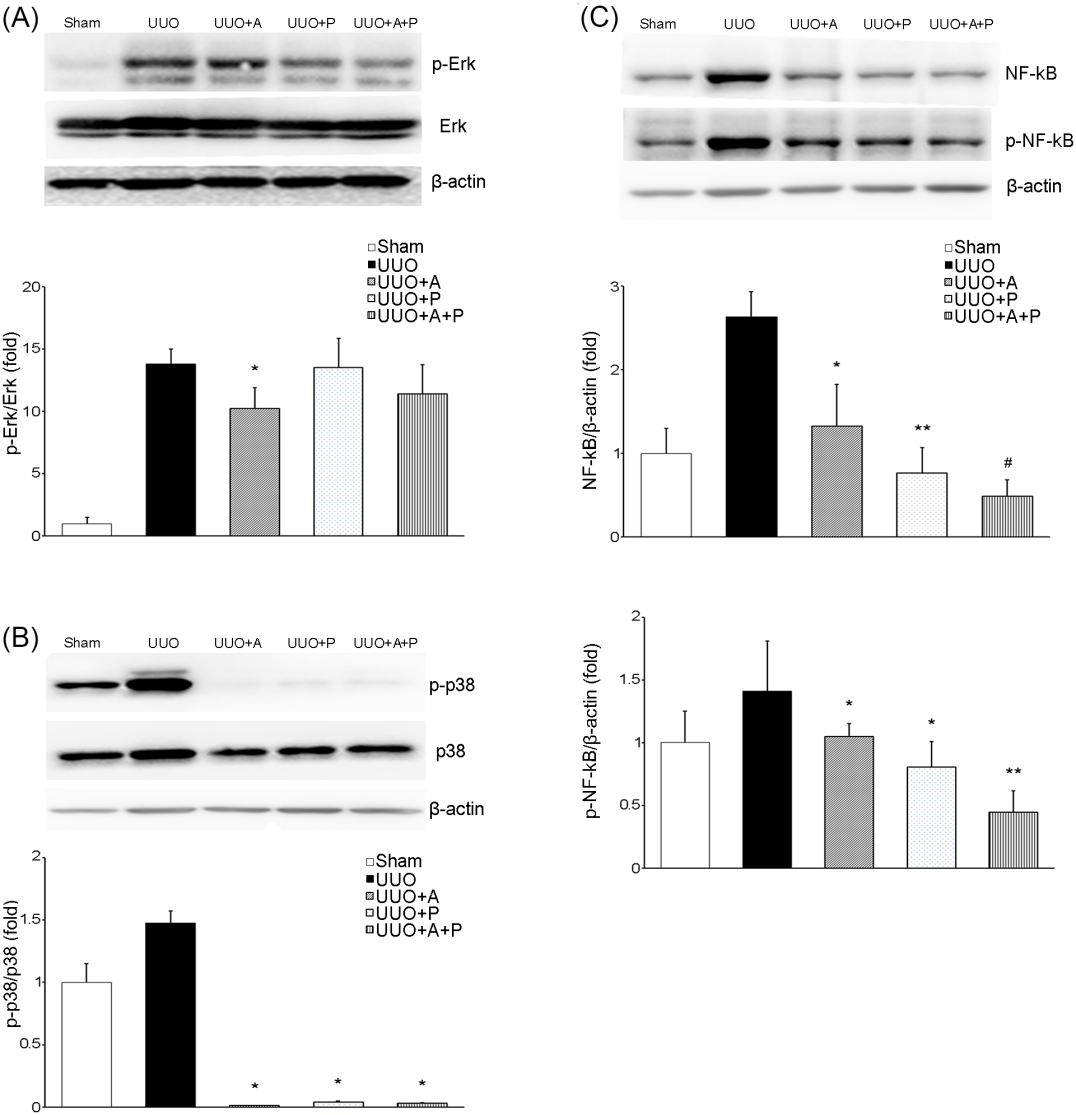
Effects of aliskiren or paricalcitol monotherapy or combination treatment with both drugs on MAPKs and NF-κB in the obstructed kidney. (A) Immunoblotting and its quantitative analysis of p-Erk and Erk showing the effect of monotherapy with aliskiren or paricalcitol or their combination in obstructive nephropathy. *P = 0.027 versus UUO. (B) Immunoblotting and its quantitative analysis of p-p38 and p38. *P<0.001 versus UUO. (C) Immunoblotting and its quantitative analysis of total NF-κB and p-NF-κB. *P<0.001 versus UUO; **P = 0.003 versus UUO+A; ^#^P<0.001 versus UUO+A and P = 0.043 versus UUO+P (for NF-κB). *P<0.001 versus UUO; **P<0.001 versus UUO+A and UUO+P (for p-NF-κB). MAPK, mitogen-activated protein kinase; NF-κB, nuclear factor kappa B; p-Erk, phosphorylated extracellular signal-regulated kinase; Erk, extracellular signal-regulated kinase; p-NF-κB, phosphorylated nuclear factor kappa B.

Phosphorylation of Erk and p38 was significantly increased in UUO kidneys, showing that the MAPK signaling pathway was activated by the UUO operation (Fig 6A and 6B). Only aliskiren monotherapy significantly attenuated the phosphorylation of Erk, and treatment combining aliskiren and paricalcitol tended to decrease the phosphorylation of Erk, but not significantly (Fig 6A). On the other hand, the combination treatment, aliskiren monotherapy and paricalcitol monotherapy markedly reduced the p-p38/p38 levels (Fig 6B). NF-κB was also obviously activated by UUO (Fig 6C and 6D). However, treatment with either aliskiren or paricalcitol significantly inhibited the UUO-induced increased expression of both NF-κB and p-NF-κB. On treating mice with a combination of aliskiren and paricalcitol, the expression of NF-κB and its phosphorylated form was more significantly decreased.

### Intrarenal renin-angiotensin system

**Fig 7:**
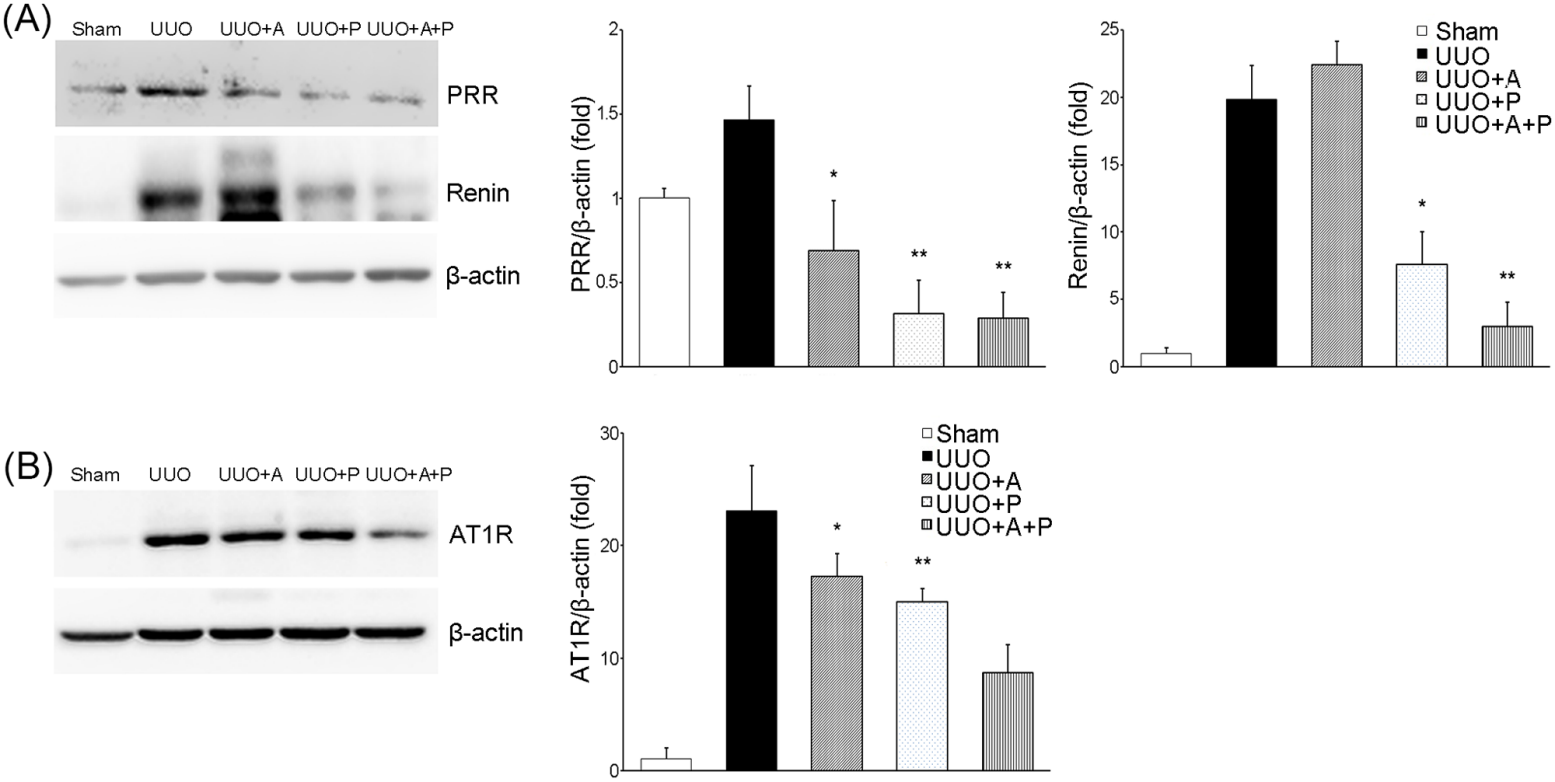
Differential effects of aliskiren or paricalcitol monotherapy or combination treatment with both drugs on the status of the renin-angiotensin system in the obstructed kidney. (A) Immunoblotting (left) and its quantitative analysis of PRR (center) and renin (right) showing the effect of monotherapy with aliskiren or paricalcitol or their combination in obstructive nephropathy. *P = 0.011 versus UUO; **P = 0.002 versus UUO and P = 0.024 versus UUO+A (for PRR). *P<0.001 versus UUO and UUO+A; **P<0.001 versus UUO and UUO+A and P = 0.001 versus UUO+P (for rennin). (B) Immunoblotting (left) and its quantitative analysis (right) of AT1R. *P<0.001 versus UUO; **P = 0.014 versus UUO+A; ^#^P<0.001 versus UUO+P. PRR, (pro)renin receptor; AT1R, angiotensin II AT1 receptor.

Western blot analysis showed that the intrarenal renin-angiotensin system was activated in the UUO control group compared to the Sham group. The amount of PRR in UUO kidneys was decreased by aliskiren monotherapy (Fig 7A). Interestingly, paricalcitol treatment more significantly reduced the expression of PRR. Treatment combining aliskiren and paricalcitol also significantly lowered the expression of PRR to the same level as paricalcitol monotherapy did. The renal concentration of renin was significantly increased in UUO kidneys and to be consistent with a previous report [6], renin concentration in UUO kidneys was slightly but not significantly increased by aliskiren monotherapy (Fig 7B). However, the renin level was significantly reduced by paricalcitol monotherapy, and it was even more substantially reduced by the combination of aliskiren and paricalcitol. The UUO-induced increased expression of AT1R was significantly attenuated by aliskiren monotherapy, and it was more significantly reduced by paricalcitol monotherapy (Fig 7C). Furthermore, combination treatment of aliskiren and paricalcitol resulted in the most significant reduction in AT1R expression.

### Renal vitamin D receptor

**Fig 8:**
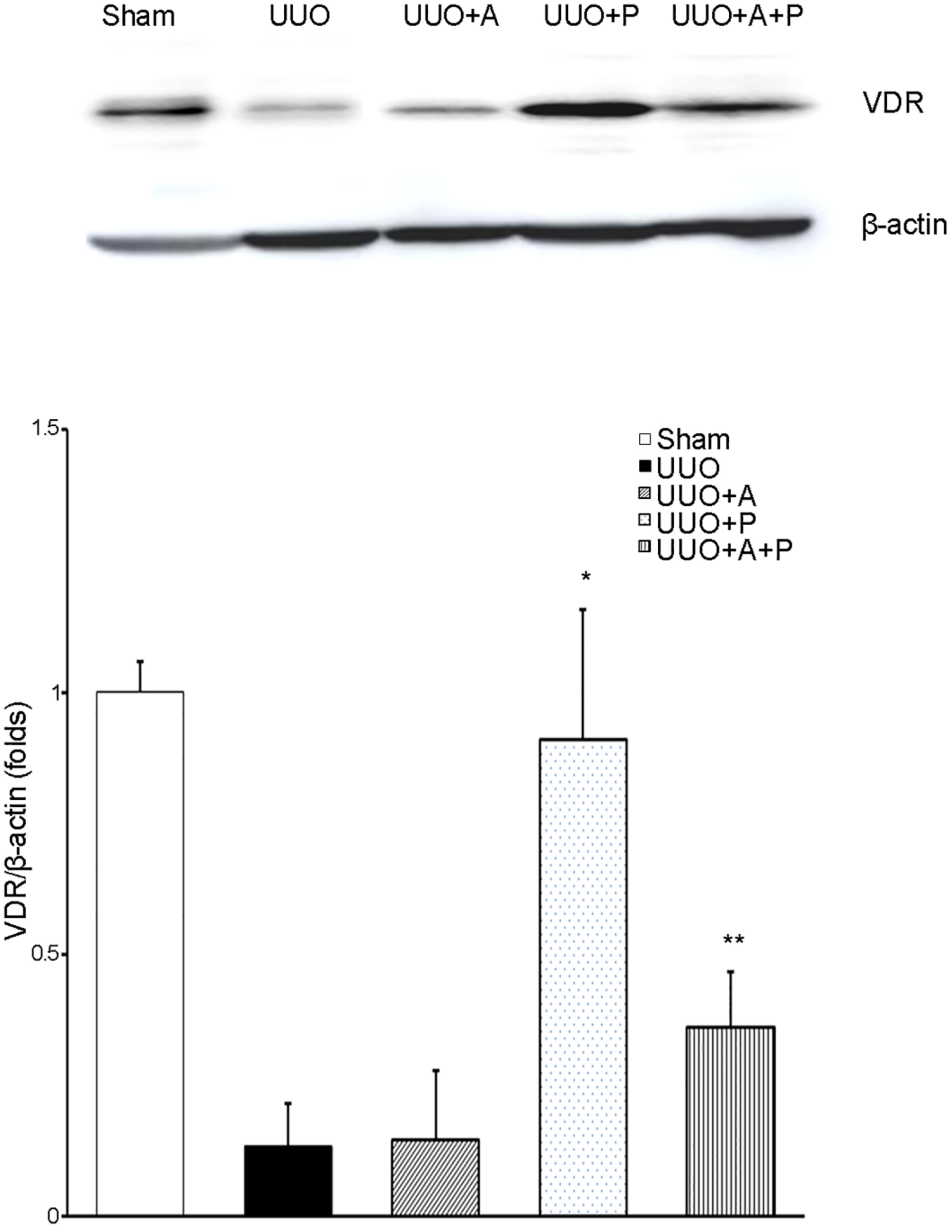
Effect of aliskiren or paricalcitol monotherapy or combination treatment with both drugs on vitamin D receptor activation in the obstructed kidney. Representative Western blot (upper) and its quantitative analysis (lower) showing VDR expression in obstructive nephropathy. *P = 0.005 versus UUO and P = 0.008 versus UUO+A and P = 0.025 versus UUO+A+P; **P = 0.040 versus UUO and P = 0.048 versus UUO+A. VDR, vitamin D receptor.

UUO caused a marked decrease in the protein expression of VDR in the kidney (Fig 8). Aliskiren monotherapy failed to affect this alteration. Paricalcitol monotherapy reversed the UUO-induced change in VDR expression in the kidney. In addition, combined treatment with aliskiren and paricalcitol significantly restored the renal VDR expression in UUO kidneys.

## Discussion

The combination of aliskiren and paricalcitol was superior to either aliskiren or paricalcitol monotherapy in attenuating progressive renal injury caused by UUO. The combined treatment more significantly reduced renal tubulointerstitial fibrosis and apoptosis. These favorable findings of the treatment combining aliskiren with paricalcitol might reflect increased protective effects against oxidative stress and inflammatory mediators. Furthermore, the beneficial effects of the combination treatment appear to be mediated largely by increased inhibition of the intrarenal renin-angiotensin system.

Induction of renal tubulointerstitial injury by UUO resulted in a state of oxidative stress, as shown by a significant increase in Nox1, Nox2, and Nox4. Aliskiren reportedly has a renoprotective effect, which is exerted through the inhibition of oxidative stress in animal models of kidney disease [25–27] and in CKD patients [28]. In doxorubicin- or streptozotocin-treated models, preventive treatment with aliskiren could significantly attenuate the increase in kidney tissue malondialdehyde (MDA) and simultaneously inhibit the decrease in superoxide dismutase (SOD), catalase and reduced glutathione in kidneys [25, 27]. In another study, aliskiren treatment attenuated glomerular superoxide in *db/db* mice, which appeared to be associated with significant suppression of NADPH oxidase activity by aliskiren [26]. Interestingly, this study found that aliskiren significantly attenuated only a specific NADPH oxidase subunit p22^phox^butitdid not affect other subunits p47^phox^ and p67^phox^ and isoforms of Nox2 and Nox4 in a type 2 diabetic animal model [26]. In the present study, UUO caused a significant elevation in levels of Nox1, Nox2, and Nox4. This difference in results of NADPH oxidase in the kidney among studies could be due in part to the difference in models of renal disease. The effect of paricalcitol on oxidative stress in models of various renal diseases has also been reported. In a rat model of amikacin-induced nephrotoxicity, renal tissue MDA levels were significantly lower, and SOD and glutathione peroxidase activities in the kidney were significantly higher when rats were treated with paricalcitol [29]. Other reports have also proven the effects of paricalcitol on renal oxidative stress biomarkers, such as thiobarbituric acid-reactive substrates and 8-hydroxy-2′-deoxyguanosine, in models of contrast- or cyclosporine-induced nephropathy [30–31]. However, a previous study using a combined aliskiren and paricalcitol treatment showed no increased effect on oxidative stress of kidneys in a streptozotocin-induced diabetic model over that of either agent alone [32]; these results are consistent with our current findings in UUO.

Either aliskiren or paricalcitol seems to be effective in decreasing renal inflammation in diseased kidneys. Levels of inflammatory markers, including expression of osteopontin, MCP-1, RANTES and tumor necrosis factor-α and staining for CD68 and F4/80, are reportedly increased in obstructed kidneys [6, 21, 33]. As shown in the current study, elevation of the intrarenal expressions of MAPKs and NF-κB is another finding of inflammation associated with UUO. Some subfamilies of MAPKs, especially Erk and p38, have been reported to be activated in response to inflammatory and stressful stimuli [34–35]. The NF-κB signaling pathway has also been associated with the regulation of many genes involved in the inflammatory response and production of inflammatory cytokines and proinflammatory mediators [34]. On the contrary, aliskiren can clearly decrease markers of macrophage infiltration in UUO kidneys [6]. In our study, aliskiren suppressed MAPKs and NF-κB, consistent with another study using an animal model harboring an artificial mouse renin gene [35]. Meanwhile, several lines of evidence have shown the potential anti-inflammatory activity of vitamin D in CKD [18–19, 36]. It has been reported that vitamin D exerts its immunomodulatory action by regulating the activity of many types of immune cells, such as macrophages, dendritic cells and T cells [18, 36]. In addition, the observed inhibitory effect of vitamin D on signaling of NF-κB in obstructive nephropathy has been attributed to the interaction of VDR and p65 NF-κB, resulting in the repression of NF-κB-mediated gene transcription [18]. Our study demonstrates for the first time that paricalcitol treatment effectively inhibits p38, but not Erk, in obstructed kidneys. Although there are few reports regarding the crosstalk between MAPKs and NF-κB, some subfamilies of MAPKs might affect NF-κB by phosphorylation of inhibitor of kappa B kinase (IKK)-α/β [34].Taken together, it appears that aliskiren and paricalcitol have similar anti-oxidant and anti-inflammatory efficacy. However, contrary to our expectations, the effects of the combined treatment in inhibiting oxidative stress or inflammation caused by UUO were not much better than those of either agent alone.

Based on our findings, it may be speculated that the treatment combining aliskiren with paricalcitol could exert more beneficial effects in preventing and attenuating tubulointerstitial fibrosis through more extensive inhibition of renin. The current study demonstrates the advantages in renal expression of PRR and AT1R in UUO mice. Despite the well-known advantages, a major drawback of adirect renin inhibitor is the compensatory increase in renin [6, 21, 32, 37]. As a result, this not only stimulates angiotensin II production [21] but also increases cellular activities through (pro)renin/PRR [1,28]. Finally, (pro)renin/PRR may induce renal fibrosis through multiple intracellular signaling pathways, either alone or in concert with activation of renal tissue RAS [1,6]. This unfavorable effect of aliskiren could be complemented by vitamin D. One of the unique properties of action of vitamin D is its ability to inhibit renin gene expression [18–19]. On a molecular level, vitamin D binds to the VDR and subsequently blocks formation of the cAMP response element (CRE)-CRE-binding protein (CREB)-CREB-binding protein (CBP) complexes in the promoter region of the renin gene, reducing its expression level [38–39]. Therefore, administration of vitamin D analogue would result in reduced activation of the RAS in a diseased kidney [18], and this finding indicates the concept that vitamin D treatment, on top of a conventional RAS inhibitor, would provide additional renoprotection [39]. To date, two studies have attempted to determine whether treatment combining aliskiren with paricalcitol may have any effect on renal injury. In a rat model of streptozotocin-induced diabetic nephropathy, aliskiren plus paricalcitol decreased renal interstitial fibrosis volume but it showed no additional effect on oxidative stress, antioxidants, renin-angiotensin system and histology of the diseased kidneys when compared to monotherapy with aliskiren or paricalcitol [32]. Another study using non-obese diabetic mice reported that the renal angiotensin I-7 and angiotensin II ratio was significantly greater in the group that received paricalcitol and aliskiren in combination without showing any histologic changes [40]. These studies used animal models of type 1 diabetes, whereas we examined the renoprotective role of the combination treatment in a non-diabetic UUO-induced kidney. Our study showed that paricalcitol provided a cushion against the compensatory increase in renal expression of renin that could be caused by individual use of aliskiren and it effectively resulted in attenuation of renal tubulointerstitial fibrosis by blocking both renin activity and its gene expression.

This study has some limitations. First, parameters of renal function were not examined. Previous studies have shown that BUN and serum creatinine were not significantly affected by UUO because of the presence of a contralateral kidney with good renal function, indicating that BUN and serum creatinine may not be good indicators of renal function in an animal model of UUO [16, 41–42]. Second, long-term efficacy and safety of combination treatment could not be evaluated in this animal model of extremely progressive nephropathy [21, 42]. Therefore, proper clinical investigations are needed to examine the transition from the experimental study to the real-world clinical practice. Finally, considering various pleiotropic effects via VDR activation [36], other potential mechanisms may explain the synergy between aliskiren and paricalcitol in protecting against renal injury. Despite these limitations, this study provides insights concerning complementary actions on molecular mechanisms associated with additional benefits from a combination of seemingly unrelated agents.

## Conclusions

The combination of aliskiren and paricalcitol increases renoprotection against non-diabetic tubulointerstitial fibrosis compared to either agent alone. This might be correlated with a more potent inhibition of the intrarenal renin-angiotensin system by the dual blockade of renin.

## Supporting information

S1 Fig The uncropped image of the gel for Fig 2A. (TIF)

S2 Fig The uncropped image for densitometric analysis of Fig 2B. (TIF)

S3 Fig The uncropped image of the gel for Nox1 in Fig 5A. (TIF)

S4 Fig The uncropped image of the gel for Nox2 in Fig 5A. (TIF)

S5 Fig The uncropped image of the gel for Nox4 in Fig 5A. (TIF)

S6 Fig The uncropped image for densitometric analysis of Nox1 in Fig 5B. (TIF)

S7 Fig The uncropped image for densitometric analysis of Nox2 in Fig 5B. (TIF)

S8 Fig The uncropped image for densitometric analysis of Nox4 in Fig 5B. (TIF)

S9 Fig The uncropped image of the gel for p-Erk in Fig 6A. (TIF)

S10 Fig The uncropped image of the gel for Erk in Fig 6A. (TIF)

S11 Fig The uncropped image for densitometric analysis of p-Erk in Fig 6A. (TIF)

S12 Fig The uncropped image for densitometric analysis of Erk in Fig 6A. (TIF)

S13 Fig The uncropped image of the gel for p-p38 in Fig 6B. (TIF)

S14 Fig The uncropped image of the gel for p38 in Fig 6B. (TIF)

S15 Fig The uncropped image for densitometric analysis of p-p38 in Fig 6B. (TIF)

S16 Fig The uncropped image for densitometric analysis of p38 in Fig 6B. (TIF)

S17 Fig The uncropped image of the gel for NF-κB in Fig 6C. (TIF)

S18 Fig The uncropped image of the gel for p-NF-κB in Fig 6C. (TIF)

S19 Fig The uncropped image for densitometric analysis of NF-κB in Fig 6C. (TIF)

S20 Fig The uncropped image for densitometric analysis of p-NF-κB in Fig 6C. (TIF)

S21 Fig The uncropped image of the gel for PRR in Fig 7A. (TIF)

S22 Fig The uncropped image of the gel for renin in Fig 7A. (TIF)

S23 Fig The uncropped image for densitometric analysis of PRR in Fig 7A. (TIF)

S24 Fig The uncropped image for densitometric analysis of renin in Fig 6C. (TIF)

S25 Fig The uncropped image of the gel for AT1R in Fig 7B. (TIF)

S26 Fig The uncropped image for densitometric analysis of AT1R in Fig 7B. (TIF)

S27 Fig The uncropped image of the gel for VDR in Fig 8. (TIF)

S28 Fig The uncropped image for densitometric analysis of VDR in Fig 8. (TIF)
