# Correction: Treatment combining aliskiren with paricalcitol is effective against progressive renal tubulointerstitial fibrosis via dual blockade of intrarenal renin

**DOI:** 10.1371/journal.pone.0196885

**Published:** 2018-05-01

**Authors:** Sungjin Chung, Soojeong Kim, Minyoung Kim, Eun Sil Koh, Seok Joon Shin, Cheol Whee Park, Yoon Sik Chang, Ho-Shik Kim

There are errors in Figs 1 and 3. The authors have provided corrected versions here.

**Fig 1:**
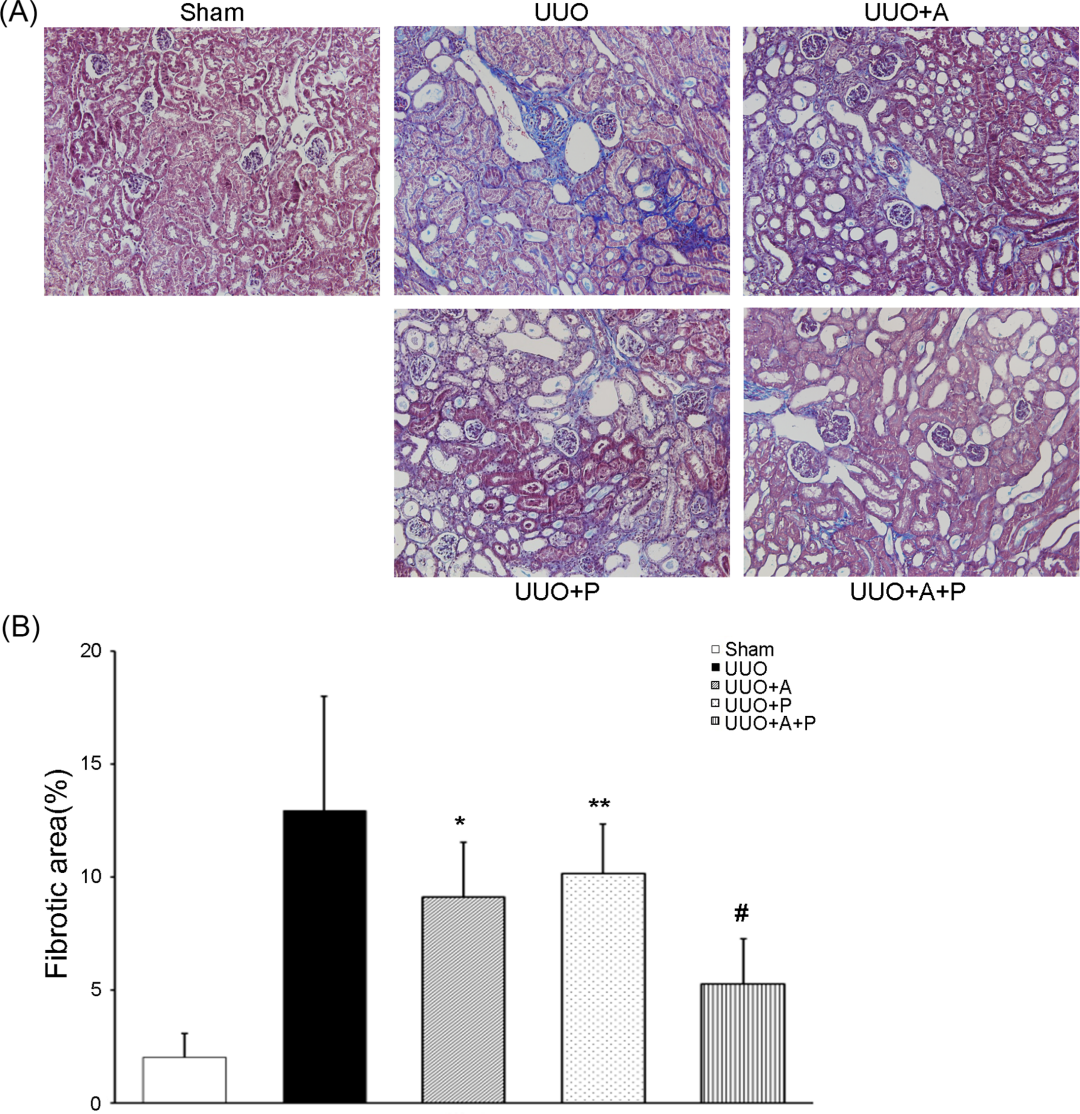
Greater beneficial effect of combination treatment with aliskiren and paricalcitol on renal fibrosis in the obstructed kidney. (A) Representative renal sections stained with Masson-trichrome (original magnifications, x200). (B) Quantitative analysis of the results for fibrotic area in the renal tubulointerstitium. *P<0.001 versus UUO; **P = 0.001 versus UUO; ^#^P<0.001 versus UUO+A or UUO+P.

**Fig 3:**
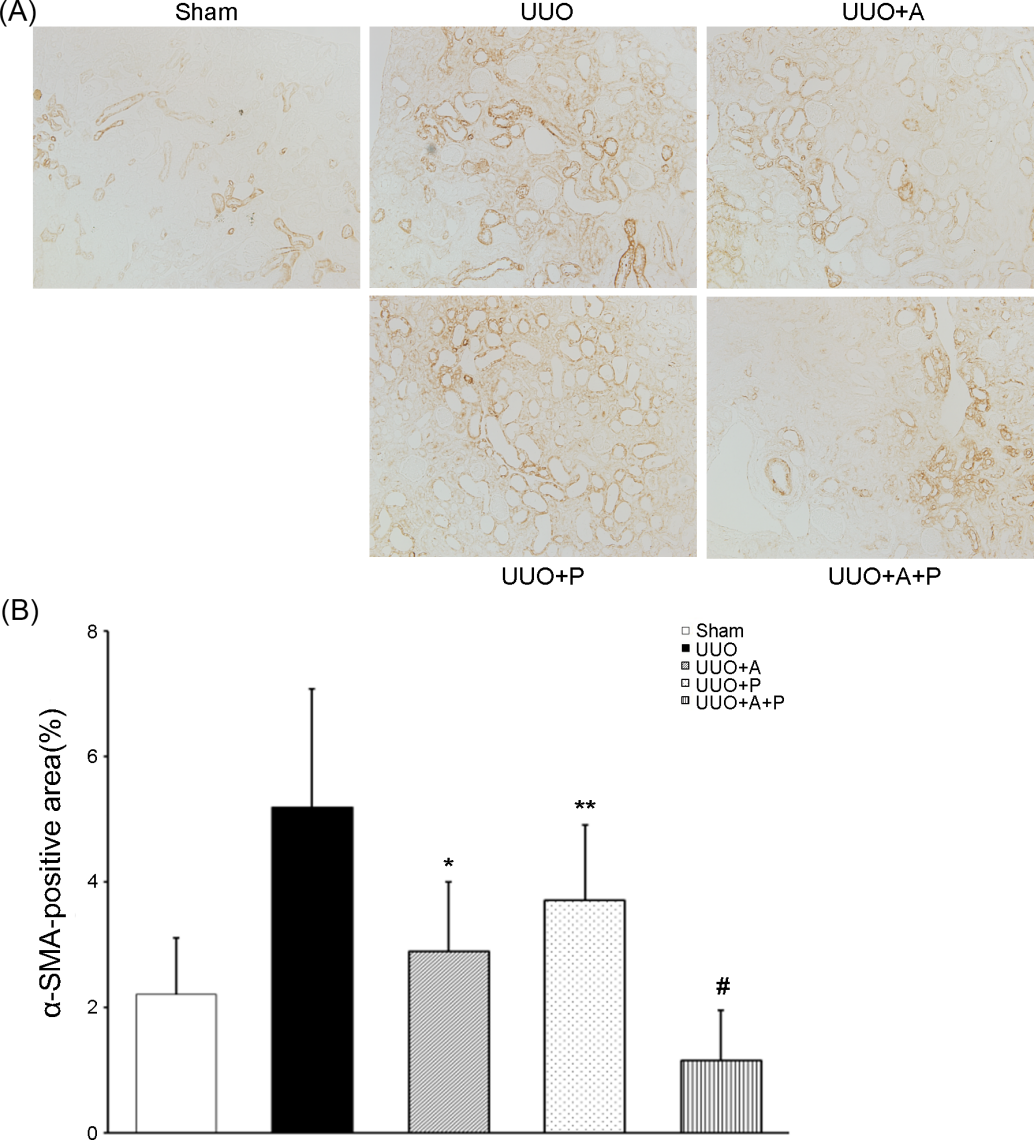
Synergistic effect of combination treatment with aliskiren and paricalcitol on the expression of myofibroblasts in the obstructed kidney. (A) Representative renal sections stained with α-SMA(original magnification, x200). (B) Quantitative analysis of the results for α-SMA in the renal tubulointerstitium. *P<0.001 versus UUO; **P = 0.001 versus UUO; ^#^P = 0.001 versus UUO+A and P<0.001 versus UUO+P. α-SMA, α-smooth muscle actin.
